# Membrane Distillation for Water Desalination: Assessing the Influence of Operating Conditions on the Performance of Serial and Parallel Connection Configurations

**DOI:** 10.3390/membranes15080235

**Published:** 2025-08-04

**Authors:** Lebea N. Nthunya, Bhekie B. Mamba

**Affiliations:** Institute for Nanotechnology and Water Sustainability, College of Science, Engineering and Technology, University of South Africa, Florida 1709, South Africa; mambabb@unisa.ac.za

**Keywords:** membrane distillation, pH stability, seawater desalination, serial and parallel module configuration, temperature polarisation

## Abstract

Though the pursuit of sustainable desalination processes with high water recovery has intensified the research interest in membrane distillation (MD), the influence of module connection configuration on performance stability remains poorly explored. The current study provided a comprehensive multiparameter assessment of hollow fibre membrane modules connected in parallel and series in direct contact membrane distillation (DCMD) for the first time. The configurations were evaluated under varying process parameters such as temperature (50–70 °C), flow rates (22.1–32.3 mL·s^−1^), magnesium concentration as scalant (1.0–4.0 g·L^−1^), and flow direction (co-current and counter-current), assessing their influence on temperature gradients (∆*T*), flux and pH stability, salt rejection, and crystallisation. Interestingly, the parallel module configuration maintained high operational stability with uniform flux and temperature differences (∆*T*) even at high recovery factors (>75%). On one hand, the serial configuration experienced fluctuating ∆*T* caused by thermal and concentration polarisation, causing an early crystallisation (abrupt drop in feed conductivity). Intensified polarisation effects with accelerated crystallisation increased the membrane risk of wetting, particularly at high recovery factors. Despite these changes, the salt rejection remained relatively high (99.9%) for both configurations across all tested conditions. The findings revealed that acidification trends caused by MgSO_4_ were configuration-dependent, where the parallel setup-controlled rate of pH collapse. This study presented a novel framework connecting membrane module architecture to mass and heat transfer phenomena, providing a transformative DCMD module configuration design in water desalination. These findings not only provide the critical knowledge gaps in DCMD module configurations but also inform optimisation of MD water desalination to achieve high recovery and stable operation conditions under realistic brine composition.

## 1. Introduction

The global demand for high-quality water is growing at an alarming rate to fulfil application requirements in various sectors, including domestic and industrial [1,2]. However, water quality is severely affected by deteriorating climate change, industrialisation and population growth [3,4]. According to the United Nations (UN) Water Reports, over two-thirds of the global population will experience clean water shortages by 2030 [5]. In response to mitigate this threatening situation, development of robust and energy-efficient desalination technologies is required to ensure water security aligned with the UN sustainability development goal (SDG-6) [6]. This has attracted research interest in alternative energy-efficient and sustainable desalination technologies, offering treatment of abundant seawater [7].

Among other technologies, membrane distillation (MD) emerged as the most promising thermally driven separation process, offering high salt rejection (>99%) and fouling tolerance [8,9,10,11]. When coupled with low-grade heat or renewable solar energy, the MD becomes economically competitive compared with reverse osmosis (RO) and multi-effect distillation (MED), especially in off-grid scenarios [8]. For instance, solar energy-powered MD consumes an energy equivalence of 1.5–3.0 kWh·m^−3^ compared to 2.5–4.0 kWh·m^−3^ and 5.0–21.5 kWh·m^−3^ in RO and MED, respectively [12,13,14]. These advantages position MD as an attractive desalination technology for use in decentralised or off-grid systems [15]. Despite the existing advantages, the MD remains critically affected by membrane wetting, temperature polarisation, concentration gradients of the modules, and inefficiencies of the system design, which impair process efficiency and therefore limit its industrial-scale deployment [16]. These challenges reduce the process driving force, affecting long-term operational performance, especially at high recoveries or high salinity conditions [17].

Research geared towards efforts to enhance the system performance of MD with a specific focus on material development, utilisation of low-grade or waste thermal energy, module configuration strategies, and process intensification to address these limitations [18,19,20]. Among these, improvements in module designs paying attention to novel flow distributions, use of spacers, and staged configurations have shown promise to enhance flux and minimise thermal polarisation [21]. Similarly, membrane module hydrodynamic arrangements focusing on flow configurations and connection architecture played a critical role in improving the system permeate flux, salt rejection, scaling/fouling resistance, and heat transfer [22,23,24]. These improvements were systematically evaluated in a single-module system to understand the influence of flow rates, temperatures, and feed salinity on process performance [25]. Although research focusing on MD dates back to the 1990s, most studies reported intensively on planar modules and flat sheet configurations, particularly at laboratory scale [26,27,28]. Comparative studies evaluating the influence of module connection configuration, including parallel and serial arrangements, under various operating conditions in DCMD remain unexplored. Optimising the architecture of module connection configuration in MD, particularly in the context of parallel and serial set-ups, plays a key role in overall process efficiency, thermal stability, and process scalability in water desalination. These configurations influence temperature gradients, differentials of vapour pressure, and therefore flux [22]. In serial connection, the feed moves through the multiple modules in series, potentially causing heat loss and reducing driving force due to cumulative thermal effects [21]. In contrast, the feed water is distributed across all modules at the same time in a parallel configuration. This maintains stable thermal gradients across modules, improving thermal efficiency [21]. Therefore, assessing the interplay between connection configuration and operating variables is essential in designing efficient and scalable MD systems for brine concentrations.

Existing reported studies often combine stages without isolating the contributions of individual module connection configurations to heat and mass transfer performance and process stability [21,29]. Therefore, the current literature has not investigated the impact of systematic parallel and serial module connection configurations on the performance of hollow fibre DCMD systems under a variety of operational conditions, including the feed temperature, flow rates, and feed chemistry. Few studies reported on pilot-scale multistage configurations without disentangling the fundamentals of mass transport and crystallisation behaviours influenced by connection architecture [30,31,32]. The current study presented the critical research gap by providing the first-of-its-kind comprehensive multiparameter evaluation of parallel and serial module connection configurations in hollow fibre DCMD. The influence of key operational variables such as feed temperatures (50, 60, and 70 °C), feed/permeate circulation (22.1, 27.5, and 32.3 mL·s^−1^), magnesium sulphate concentration as a scalant (1.0, 2.0, and 4.0 g·L^−1^), and co-current vs. counter-current flow orientation was evaluated, addressing the major research gaps in understanding the module configuration interactions in DCMD systems. The feed solution was composed of 3.5 wt.% NaCl and varying MgSO_4_ concentrations. Also, the process optimisation uncovered the impact of process parameters on performance indicators such as permeate flux, thermal gradients (∆*T*), conductivity, and pH profiles, thus underpinning the interplay between module configurations and process stability.

The novelty of this study lies in the fundamental understanding of hollow fibre module connection configurations with integrated performance assessment correlating to permeate flux, thermal gradients, feed and permeate conductivity profiles, pH evolution, and crystallisation behaviour of produced salts. This advanced the knowledge in demonstrating the influence of module connection strategies on polarisation, crystallisation dynamics, and wetting behaviour of the hollow fibre membrane, even under identical conditions. The experimental design unpacked the manifestation of temperature gradient collapse influenced by flow distribution regimes. Also, the connection configuration presented a direct impact on stepwise thermal and concentration gradients, which can be beneficial for salt recovery. Therefore, the study highlighted the holistic evaluation of the interplay between membrane module connection configurations and multiparameter operational regimes in hollow fibre DCMD, offering a new perspective in seawater desalination.

## 2. Materials and Methods

### 2.1. Materials

Magnesium sulphate (MgSO_4_, 98% ACS reagent) and sodium chloride (NaCl, 99% ACS reagent) were purchased from Sigma Aldrich (Schnelldorf, Germany). These salts were used to prepare 3.5 wt.% NaCl seawater spiked with varying concentrations of MgSO_4_ as a scalant (0.5, 1.0, 2.0, and 4.0 g·L^−1^). The deionised water was prepared in our lab using the Direct-Q^®^ (Millipore) system supplied by Merck Millipore (Modderfontein, South Africa). The hollow fibre polypropylene (PP) membrane (MD 020 CP 2N) with average pore size (0.2 µm), inner diameter (1.8 mm), membrane surface area (0.1 m^2^), 40 capillaries, length (500 mm), and porosity (73%) was purchased from Microdyn-Nadir GmbH (Wiesbaden, Germany).

### 2.2. Membrane Distillation System Setup

**Figure 1:**
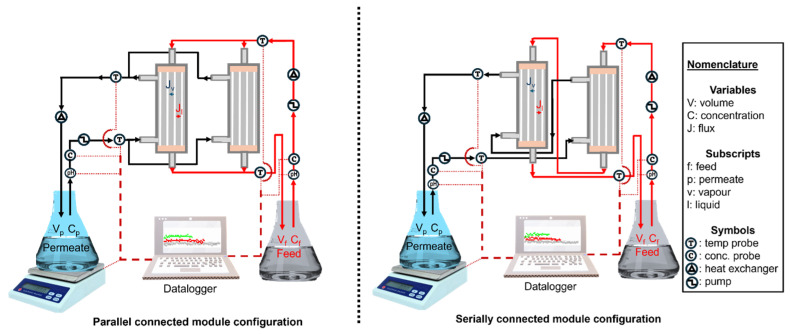
Schematic illustration of parallel and serially connected module configurations. Hollow fibre membranes were assessed in direct contact membrane distillation. The red process streams connecting the membrane module and the tank represented the feed flow while the black streams represented the permeate flow.

Two hollow fibre membrane modules operated in direct contact membrane distillation (DCMD) were configured in parallel and serial connection (Figure 1) to assess their impact on process performance during water extraction from synthetic brine (3.5 wt.% NaCl, spiked with 0.5 g·L^−1^ MgSO_4_). All membrane modules were placed in a vertical orientation, with the feed flowing downwards to minimise accumulation of crystallised salts and possible blockage of the membrane channels. A parametric design focusing on the independent one-factor-at-a-time (OFAT) model was used to isolate the influence of each parameter on process performance. This model ensured high-quality data without confounding interactions, which are influenced by the complexity of independent thermal and mass transfer mechanisms in DCMD. Assessed parameters were feed temperature (50, 60, and 70 °C), permeate/feed circulation flow rate (22.1, 27.5, and 32.3 mL·s^−1^), varying concentration of MgSO_4_ (1.0, 2.0, and 4.0 g·L^−1^) and crossflow configuration (counter-current and co-current). The permeate temperature was kept constant at 15 °C. The feed and permeate inlet and outlet temperatures corresponding to each set feed temperature of 50, 60, and 70 °C are presented in Table 1. These operational parameters were selected based on their direct influence on hollow fire DCMD systems. The chosen flow rates of 22.1, 27.5, and 32.3 mL·s^−1^ correspond to linear velocities of 0.440, 0.547, and 0.642 m·s^−1^ from the tubing of 8.0 mm diameter. These flow rates provided suitable flow conditions corresponding to Reynolds numbers of 736–1070. These flow rates represented realistic and literature-supported hydrodynamic conditions and flow regimes for hollow fibre MD, which enabled shear-based mass transport without risking the integrity of the membrane or causing pore wetting, thus compromising salt rejection process performance [33,34]. The feed temperature (50–70 °C) and MgSO_4_ concentrations (1.0–4.0 g·L^−1^) represented the possible solar energy input with high driving force and pre-scaling conditions, commonly found in seawater, respectively. The permeate weight changes were measured using a equipment LBX 3 weighing balance (Adam Equipment S.A. PTY, Johannesburg, South Africa) to evaluate the permeate flux, where Equation (1) was used. Furthermore, the permeate/feed conductivity and pH were monitored using a HI-5522-02 Professional benchtop pH/ISE/EC dual channel meter (Hanna Instruments, Johannesburg, South Africa) to assess the process salt behaviour, acid-base equilibrium, and membrane selectivity (rejection, which was evaluated from Equation (2)). To assess the temperature difference (∆*T*) across the membrane interfaces, the feed and permeate temperatures were measured at the inlet and outlet of modules configured in parallel and series using a Techme temperature LCD digital thermometer (SabiNano PTY, Johannesburg, South Africa). These measured inlet and outlet point temperatures were used to evaluate the ∆*T* fluctuations across the membrane following Equations (3)–(5). The supersaturated brine with 75% water recovery was filtered to recover crystal salts, which were dried at 60 °C for 24 h.

(1) $$J=\frac{dm}{Adt}$$

(2) $$R\left(\%\right)=\frac{k_{f}-k_{p}}{k_{f}}\times 100\%$$

(3) $${\Delta T}_{In}=\frac{{\Delta T}_{in}-{\Delta T}_{out}}{In\frac{{\Delta T}_{in}}{{\Delta T}_{out}}}$$

(4) $${\Delta T}_{in}=T_{feed,\;in}-T_{permeate,out}$$

(5) $${\Delta T}_{out}=T_{feed,\;out}-T_{permeate,in}$$

where *J*, *R*(%), *dm*, *A*, *dt*, *k_f_*, and *k_p_* represent water flux, percentage salt rejection, mass difference at time (*t*), change in time, feed, and permeate conductivity, respectively.

## 3. Results and Discussion

### 3.1. Effect of Feed Temperature (50, 60, 70 °C)

**Figure 2:**
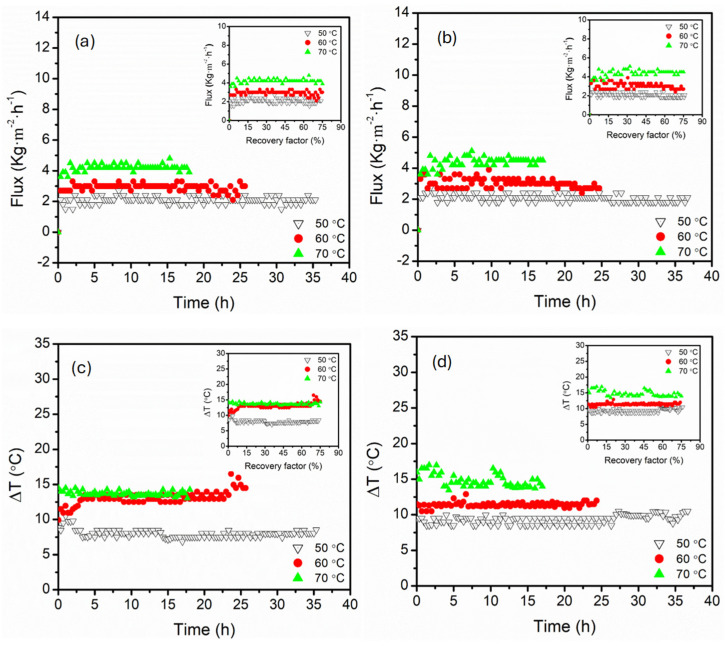
The evolution of permeate flux for (**a**) parallel and (**b**) serial hollow fibre DCMD module configurations, and transmembrane temperature difference (∆*T*) for (**c**) parallel and (**d**) serial configurations under varying feed temperatures (50, 60, and 70 °C). Plot insert presented corresponding flux and ∆*T* as a function of recovery factor. The feed and permeate circulation flow rate and MgSO_4_ concentrations were kept constant at 17.5 mL·s^−1^ (0.35 m·s^−1^) and 0.5 g·L^−1^, respectively. The DCMD was operated in a counter-current configuration.

The influence of feed temperature on the permeate flux behaviour and temperature difference (∆*T*) profiles for parallel and serial module configurations is presented in Figure 2. Like existing literature, increased feed temperature enhanced permeate flux for both configurations. This was associated with an exponential increase in vapour pressure induced by increased ∆*T*, which served as the primary driving force in [35,36,37]. In parallel connection, the flux remained relatively steady across the entire duration of the experiment, especially at 60 and 70 °C, ensuring flux stabilisation around 3.0 and 4.2 kg·m^−2^·h^−1^, respectively, even at high recovery factors above 74% (Figure 2). Parallel connection maintained relatively stable ∆*T*, ranging from 7.5, 13.2, and 14.0 °C for 50, 60, and 70 °C feed temperatures, respectively. As a result, a consistent driving force was maintained across the modules in parallel due to symmetric and uniform heat and mass transfer profiles. This configuration reduced the loss of thermal energy along the membrane length, resulting in favourable ∆*T* and stable fluxes even at high recovery factors (≤74%). Although not directly calculated, it is important to understand that reduced flow velocity caused by water splitting in parallel modules likely decreased Nusselt numbers (Nu), thus affecting convective heat transfer and contributing to the reported stable ∆*T* [38,39]. Conversely, the serial connection exhibited flux fluctuations, particularly at 60 °C. These fluctuations were more pronounced at low recovery factors, which were influenced by the sequential heat exchange across the modules in series [40]. The feed solution experienced significant cooling at the first module, reducing the driving force, particularly at 70 °C. This was evidenced by a large ∆*T* exceeding 16.9 °C at the early stage of operation, indicative of a strong driving force. However, ∆*T* decreased steadily with time, highlighting the impact of heat dissipation across serial modules. This degradation reflected the thermal vulnerability of serial configurations, where the second module operated under compromised vapour pressure due to heat losses upstream. Also, flux and ∆*T* fluctuations seen in serial connection implied higher module connection sensitivity to temperature polarisation effects. Interestingly, both configurations demonstrated stable flux (2.1–2.4 kg·m^−2^·h^−1^) and ∆*T* (8.0–8.4 °C) at low feed temperatures (50 °C). The stability at this feed temperature reflected maintenance of thermal driving force across both module configurations due to minimised internal temperature decay.

**Figure 3:**
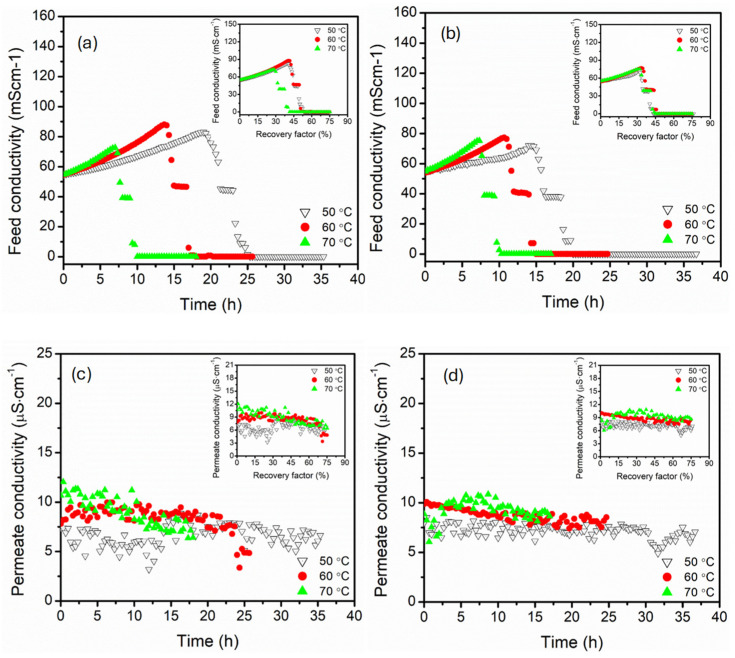
The feed conductivity profiles of the (**a**) parallel and (**b**) serial configurations and the permeate conductivity profiles of the (**c**) parallel and (**d**) serial configurations, all versus time in hollow fibre DCMD at feed temperatures of 50, 60, and 70 °C. Plot insert reflected the corresponding conductivity trends as a function of recovery factor. The feed and permeate circulation flow rate and MgSO_4_ concentrations were kept constant at 17.5 mL·s^−1^ (0.35 m·s^−1^) and 0.5 g·L^−1^, respectively. The DCMD was operated in a counter-current configuration.

The feed conductivity profiles reflected the progressive accumulation of the salts for both parallel and serial module configurations due to water extraction from the feed. The feed conductivity increased with time, consistent with rising solute concentrations during the system advancement to higher recovery factors (Figure 3). In parallel configuration, the feed conductivity increased gradually with a stable rise, particularly at 70 °C, suggesting efficient water vapour extraction with minimal membrane scaling. The insert plot confirmed a linear relationship between feed conductivity and recovery factor, indicating a predictable rise in feed concentrations. The feed conductivity suddenly declined after 6 h of operation, likely due to salt precipitation (crystallisation) at high recovery factors (>30%). The sharp decline in feed conductivity was attributed to progressive removal of the water from the feed, thus concentrating the solution to supersaturation. Upon continuous water extraction from the feed, crystallisation was triggered, forming salt. This process reduced the number of free mobile ions in the bulk feed, causing an abrupt decline in conductivity [41,42]. At low feed temperatures (60 and 50 °C), the flux decline occurred after 15 and 20 h of operation, possibly due to the slow rate of water recovery (small flux). In the serial configuration, the rate of conductivity followed a similar trend, though with a sharp rise at the initial stage for 70 °C. This reflected aggressive vapour extraction as evidenced in flux data, demonstrating rapid concentration of the feed. A similar sharp drop in feed conductivity was recorded after a long duration of operation (6 h), potentially indicating intense salt crystallisation driven by degradation of solubility limits along the series of membranes [42]. 60 °C feed temperature demonstrated limited feed conductivity variation for both parallel and serial module configurations. However, lower feed temperature (50 °C) presented significantly different durations of conductivity decline, where a sharp decline was recorded after 16 h of operation in serial connection compared to 20 h in parallel connection. The differences were associated with solute solubility, which was affected by the reduced bulk temperature of the feed along the serial module configuration [23]. Based on these results, serial connection promoted a rapid rise in feed conductivity, especially at higher feed temperatures, which induced an earlier onset of crystallisation due to localised supersaturation. On one hand, the parallel configuration presented stable, controlled solute accumulation dynamics. The permeate conductivity provided the membrane selective recovery of high-quality water while exclusively retaining the solutes. The permeate conductivity of all configurations was maintained below 20 µS·cm^−1^ for all feed temperatures, indicating salt rejection exceeding 99%. The permeate conductivity decreased gradually for both configurations as the recovery factors increased, likely due to dilute effects caused by the extraction of more water while ensuring enhanced salt retention. The most stable values were recorded at 60 °C feed temperature, though 70 and 50 °C feed temperatures still maintained excellent salt rejection (>99%), albeit with more fluctuations due to thermal stress on the membrane interface and pores. Higher and more variable permeate conductivities were recorded in parallel connection, particularly at 70 °C. Intense fluctuation of permeate conductivity at 70 °C was associated with concentrations of polarisation effects along the serial module connection, increasing the risk of pore wetting [43]. Nonetheless, the average permeate conductivity remained within acceptable limits of water desalination, underscoring the robustness of both parallel and serial membrane configuration systems.

**Figure 4:**
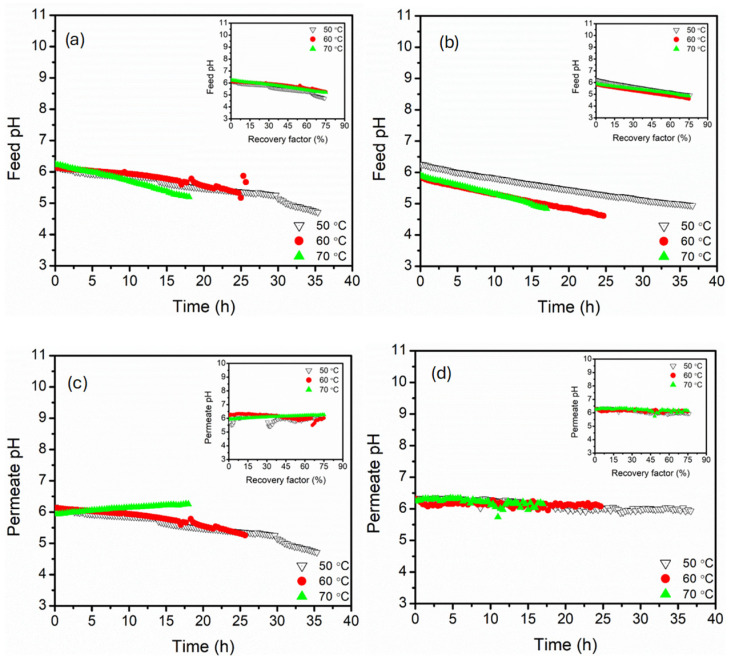
The feed pH profiles of (**a**,**b**), parallel and serial module configurations, permeate pH profiles of (**c**,**d**), parallel and serial module configurations. The experiments were conducted at feed temperatures of 50, 60 and 70 °C. The pH was recorded as a function of time, and a plot insert recording pH as a function of recovery factor. The feed and permeate circulation flow rate and MgSO_4_ concentrations were kept constant at 17.5 mL·s^−1^ (0.35 m·s^−1^) and 0.5 g·L^−1^, respectively. The DCMD was operated in a counter-current configuration.

The pH profiles of the feed and permeate recorded against time were provided to highlight the membrane chemical stability and ion rejection behaviours under different feed temperatures and module connection configurations (Figure 4). The influence of recovery factors was provided as a plot insert, highlighting the impact of water recovery on feed and permeate pH. Spiking the feed with 2 g·L^−1^ in addition to 3.5% NaCl reduced the solution pH to approximately 6.4. The acidification of the feed was associated with the hydrolysis of the Mg^2+^ ions and acidic characteristics of MgSO_4_ in aqueous streams [44]. Contrary to typical single NaCl rejection systems, the feed pH decreased with time and recovery factors (plot insert) for both parallel and serial module configurations, with the rapid decline at high feed temperature (70 °C). The decrease in pH was associated with progressive concentration of MgSO_4_ caused by water extraction from the feed, intensifying accumulation of acidic species such as H^+^ and HSO_4_^_^ ions. The solution equilibrium shifted to a lower pH. The serial connection configuration presented a more pronounced pH decline compared to the parallel set-up. This was associated with intensified concentration polarisation along the axial flow on serial modules, establishing locally intensified ionic strength and pH depression zones [45]. In parallel configuration, stable pH decline was recorded, likely due to the distribution of feed flow across parallel modules, reducing accumulation of MgSO_4_ and delayed acidification. Therefore, the parallel module configuration enhanced buffering capacity against pH collapse compared to the serial configuration, which was more sensitive to cumulative concentration effects. The permeate pH of both configurations remained relatively stable (6.0–6.5), with mild changes (±0.5) at high feed temperatures. The pH stability of the permeate affirmed non-volatile acidic species, which were effectively rejected by the hydrophobic PP hollow fibre membrane [46]. Despite salt crystallisation evidenced by abrupt feed conductivity decline at high recovery factors, the permeate pH stability was influenced by the absence of significant acid/base equilibria as the salts precipitated. However, a slight decline in parallel configuration at low feed temperature (50 and 60 °C) was attributed to partial membrane wetting, encouraging transport of dissolved acidic species [47].

### 3.2. Effect of Circulation Flow Rate (22, 27, 32 mL·s^−1^)

**Figure 5:**
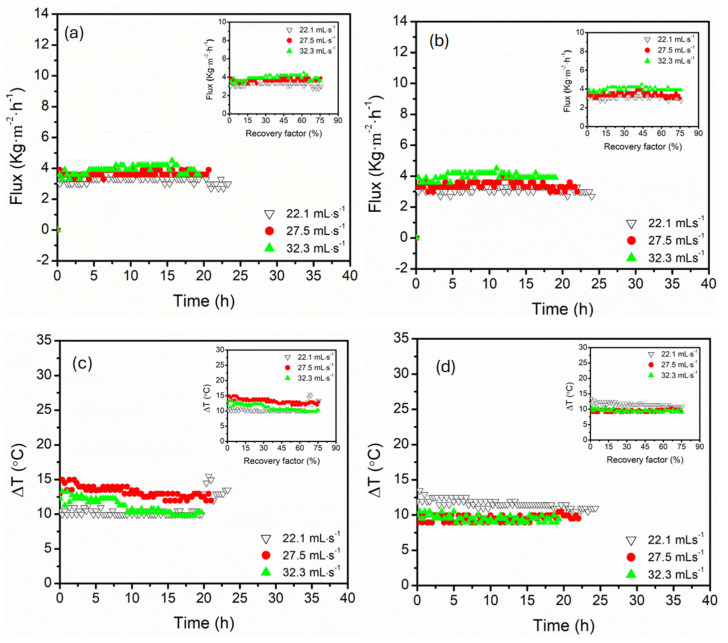
The evolution of permeate flux for (**a**) parallel and (**b**) serial hollow fibre DCMD module configurations, and transmembrane temperature difference (∆*T*) for (**c**) parallel and (**d**) serial configurations under varying feed flow rates (22.1, 27.5, and 32.3 mL·s^−1^). Insert presented corresponding flux and ∆*T* as a function of recovery factor. The feed was composed of 3.5 wt.% NaCl and 0.5 g·L^−1^ MgSO_4_. The feed temperature was kept constant at 60 °C. The DCMD was operated in a counter-current configuration.

The influence of feed and permeate circulation flow rates on the rate of water recovery (permeate flux) and temperature polarisation (indicated by ∆*T*) was assessed systematically for parallel and serial hollow fibre DCMD configurations (Figure 5). Three tested flow rates were 22.1, 27.5, and 32.3 mL·s^−1^ at a fixed feed composition of 3.5 wt.% NaCl, 0.5 g·L^−1^ MgSO_4_, 60 °C and 15 °C feed and permeate temperatures, respectively. Generally, the permeate flux increased with flow rate for both configurations due to a reduction in the layer thickness of the thermal boundary layer and enhancement of heat and mass transfer coefficients. Also, increased linear velocity at high flow rates reduced temperature polarisation, promoting high driving force. The flux increased more sharply with an increase in feed flow rate in the serial configuration. The flux was maintained at 4.2 kg·m^−2^·h^−1^ upon increasing the flow rate to 32.3 mL·s^−1^. However, a slight decline was recorded at recovery factors above 56% (see plot insert). In parallel configuration, the increase in flow rate, particularly 32.3 mL·s^−1^, demonstrated moderate flux enhancement, stabilising at 3.9 kg·m^−2^·h^−1^. The lower flux enhancement in parallel configuration was associated with non-uniform flow distribution across modules, resulting in poor alignment with pressure drop, which limited uniform shear and temperature gradients across the membrane [21,48]. It is worth noting that flux enhancement influenced by increased flow rates was more subdued compared to the exponential increase reported in feed temperature variations. Unlike thermal driving force, the flow-dependent improvements were controlled by hydrodynamics and boundary layer, resulting in skewed flux enhancements [10].

∆*T* presented an inverse correlation with an increase in flow rate, suggesting improvement in convective heat transfer and reduced thermal loss along the module [49,50]. In serial connection, ∆*T* was relatively stable, ranging between 8.9 and 9.4 °C at higher flow rates (32.3 and 27.5 mL·s^−1^). ∆*T* stability suggested thermal efficiency of the system with minimal temperature polarisation along the sequential flow. System efficiency was associated with cumulative heating along the serial module connection, sustaining a favourable temperature gradient [51]. ∆*T* exhibited greater variability in parallel configuration, ranging from 12.2 to 14.9 °C. This was attributed to thermal gradient disparity within individual modules, causing inefficient thermal coupling [52].

**Figure 6:**
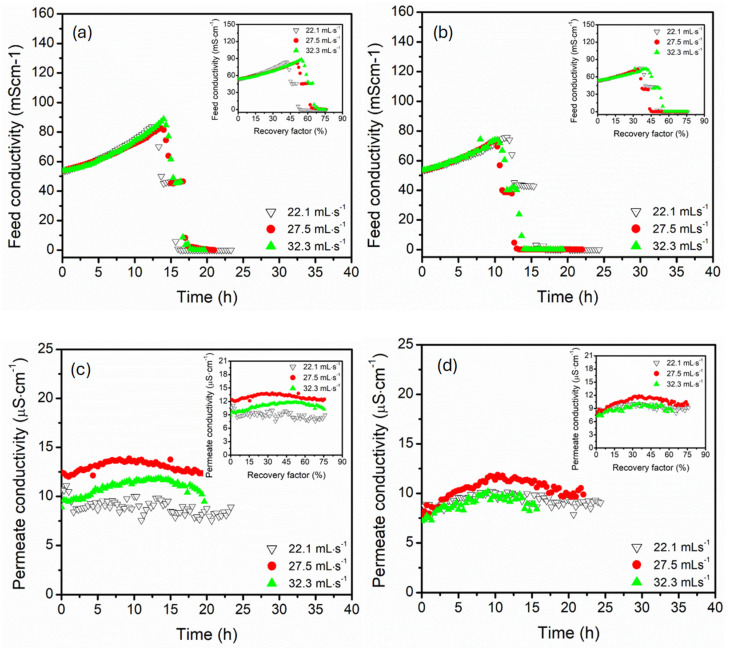
The feed conductivity profiles of the (**a**) parallel and (**b**) serial configurations and the permeate conductivity profiles of the (**c**) parallel and (**d**) serial configurations, all versus time in hollow fibre DCMD at circulation flow rate of 22.1, 27.5, 32.3 mL·s^−1^. Plot insert reflected the corresponding conductivity trends as a function of recovery factor. The feed was composed of 3.5 wt.% NaCl and 0.5 g·L^−1^ MgSO_4_. The feed temperature was kept constant at 60 °C. The DCMD was operated in a counter-current configuration.

Figure 6 presents the collective influence of module connection configuration and feed/permeate circulation flow rate (22.1, 27.5, and 32.3 mL·s^−1^) on the dynamic ionic, with specific implications for process stability and salt precipitation at high recovery factors. Both configurations presented an initial increase in feed conductivity with operation time, which is caused by progressive water extraction resulting in the concentration of NaCl and MgSO_4_ salts in the feed stream. This trend was higher at lower flow rates (22.1 mL·s^−1^) due to minimal convective mixing, favouring accumulation of formed salts. Increasing the circulation flow rate to 32.3 mL·s^−1^ enhanced the feed turbulence, resulting in delayed salt precipitation. The increase in feed concentration was rapid in a parallel configuration, yielding the peak conductivity of 85 mS·cm^−1^ at a recovery factor range of 45–57%, followed by an immediate decline. The conductivity reversal at high recovery factors was influenced by supersaturation and crystallisation of the feed solution. The solubility limit of the salts reduced the concentration of dissolved ions, decreasing the conductivity [53,54,55]. The serial configuration presented the gradual decrease and delayed onset of feed conductivity decline, which was influenced by concentration build-up across modules in series and the slow rate of water recovery, particularly at low flow regimes. The permeate conductivity remained relatively stable for all configurations and circulation flow conditions, with the maximum conductivity of 13.9 µS·cm^−1^, demonstrating salt rejection of >99.9%. The permeate conductivity was slightly elevated at high feed flow rates (27.5 and 32.3 mL·s^−1^), particularly in parallel configurations, suggesting the onset of membrane wetting and concentration polarisation, causing stress on the membrane pores [10]. Under low flow conditions (22.1 mL·s^−1^), the lowest conductivity was achieved, underscoring the beneficial role of lower flow rates to enhance membrane selectivity, particularly in parallel configurations. However, the opposite was true in serial configuration, where increased flow rates (32.3 mL·s^−1^) resulted in decreased permeate conductivity due to reduced concentration polarisation effects and minimal salt stress on the membrane pores. In summary, the feed/permeate flow rates emerged as critical operational parameters, influencing the balance between flux, salt rejection, and salt crystallisation control. High flow rates delayed the crystallisation and maintained salt rejection, particularly in the serial module configuration. Comparing the two configurations, the parallel module connection experienced abrupt conductivity shifts, while the serial connection exhibited stable patterns, especially under high flow conditions.

**Figure 7:**
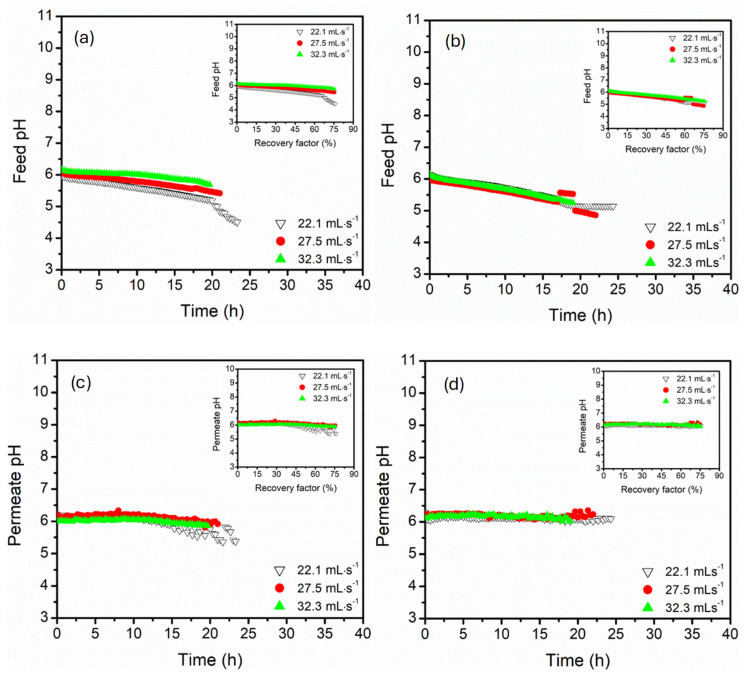
The pH profiles of the feed (**a**) parallel and (**b**) serial hollow fibre DCMD, permeate in (**c**) parallel and (**d**) serial hollow fibre DCMD under varying feed and permeate circulation flow rate (22.1, 27.5, 32.3 mL·s^−1^). The feed was composed of 3.5 wt.% NaCl and 0.5 g·L^−1^ MgSO_4_. The feed temperature was kept constant at 60 °C. The DCMD was operated in a counter-current configuration.

The influence of three water circulation rates (22.1, 27.5, and 32.5 mL·s^−1^) on the pH evolution of the feed and permeate was assessed in both parallel and serial module configurations. The feed pH exhibited a progressive decline over time across all tested conditions (Figure 7). Acidification of the feed water decline was pronounced in a parallel configuration due to localised acidification, which correlated with high thermal polarisation (∆*T*) and lower flux. The feed pH dropped to approximately 4.5, particularly at low flow rates (22.1 mL·s^−1^), which was caused by limited convective mixing amplifying the ionic speciation shift near the membrane interface. The feed pH of the serial configuration decreased gradually, reaching 5.0 even at high recovery factors (75%). This stability was explained by improved hydrodynamics of dilute accumulated acidic species. The permeate pH remained relatively stable under all tested conditions, indicating minimal transport of acid/base species through the membrane. Notably, the flux fluctuations were better preserved in the serial configuration compared to the parallel. This was explained by back-diffusion or localised membrane wetting, which facilitated transport of acid species [43,56,57].

### 3.3. Effect of Magnesium Sulphate Concentration (1.0, 2.0, 4.0 g·L^−1^)

**Figure 8:**
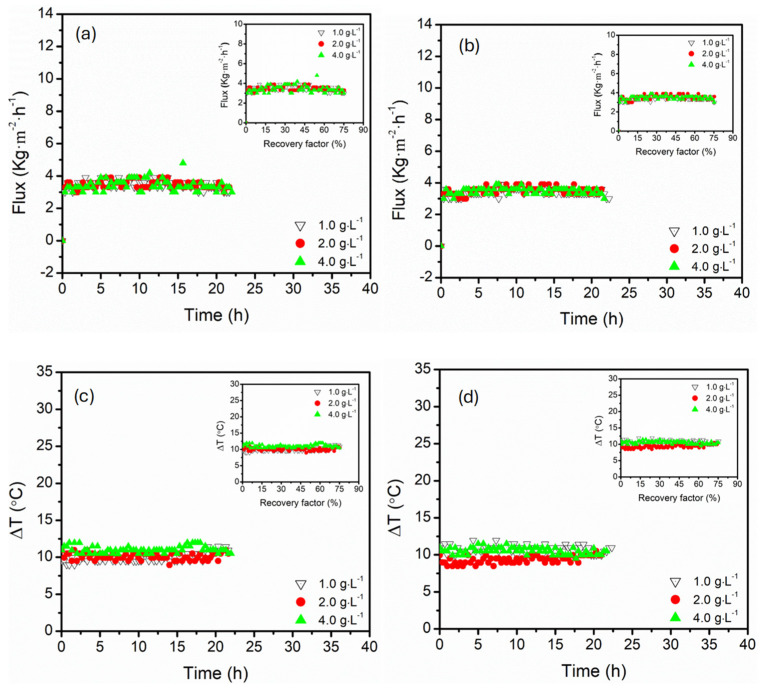
The evolution of permeate flux for (**a**) parallel and (**b**) serial hollow fibre DCMD module configurations, and transmembrane temperature difference (∆*T*) for (**c**) parallel and (**d**) serial configurations under varying MgSO_4_ (1.0, 2.0 and 4.0 g·L^−1^). Insert presented corresponding flux and ∆*T* as a function of recovery factor. The feed and permeate circulation flow rate and feed temperature were kept constant at 27.5 mL·s^−1^ (0.547 m·s^−1^) and 60 °C, respectively. The DCMD was operated in a counter-current configuration.

The operation stability of MD systems is critically influenced by the solute concentrations, particularly sparingly soluble salts such as MgSO_4_. In the current study, the influence of MgSO_4_ concentrations (1.0, 2.0, and 4.0 g·L^−1^) on permeate flux and temperature difference (∆*T*) was assessed across both parallel and serial membrane module configurations, where feed temperature and flow rates and NaCl concentration were fixed at 60 °C, 27.5 mL·s^−1^, and 3.5 wt.%, respectively (Figure 8). The permeate flux remained relatively stable across all tested MgSO_4_ over the entire operational period (22.5 h), with slight variations and no significant decline associated with membrane scaling. The apparent resistance to scaling, particularly at high MgSO_4_ concentration (4.0 g·L^−1^), was associated with short duration of operation (~23.0 h), high flow rates, and preferential optimum feed temperature (50–70 °C). These factors prevented MgSO_4_ built-up on the surface of the membrane. Although flux and ∆*T* stability with negligible decline was maintained even at high MgSO_4_ concentration (4.0 g·L^−1^), suggesting minimal performance loss, conclusive evidence of MgSO_4_ scaling would require membrane analysis (SEM, EDS and energy of interaction between membrane and scalant) [58], which was not part of this study. Therefore, resistance to scaling was rather based on operational stability under the tested conditions. Similarly, serial connection offered marginally stable flux profiles, especially for high MgSO_4_ concentrations. This was explained by the distributed architecture of vapour extraction in serial arrangement, managing the salt concentration built-up and scale formation [22]. In parallel configuration, the driving force demand was concentrated across both modules simultaneously, amplifying sensitivity of the salt build-up, particularly when MgSO_4_ concentration exceeded 2.0 g·L^−1^. Similarly, ∆*T* remained relatively stable (approximately at 9.4–10.9 °C) for both parallel and serial module configurations, indicating process resistance to thermal fouling or scaling-induced thermal degradation. Resistance to ∆*T* variation indicated that the thermal conductivity of the feed was not altered within the tested MgSO_4_ concentration. Importantly, the circulation flow rate (27.5 mL·s^−1^) was sufficient to suppress the thermal boundary layer expansion as the solute concentration accumulated with an increase in recovery factor, resulting in homogenised temperature difference [59]. Notably, ∆*T* was more stable in serial configuration, particularly at 4.0 g·L^−1^ MgSO_4_ concentration, suggesting uniform heat distribution along the module length, causing thermal resilience. This aligned with permeate flux under tested MgSO_4_ conditions, confirming the thermal and mass transfer advantage of the serial connection under high salinity regimes.

**Figure 9:**
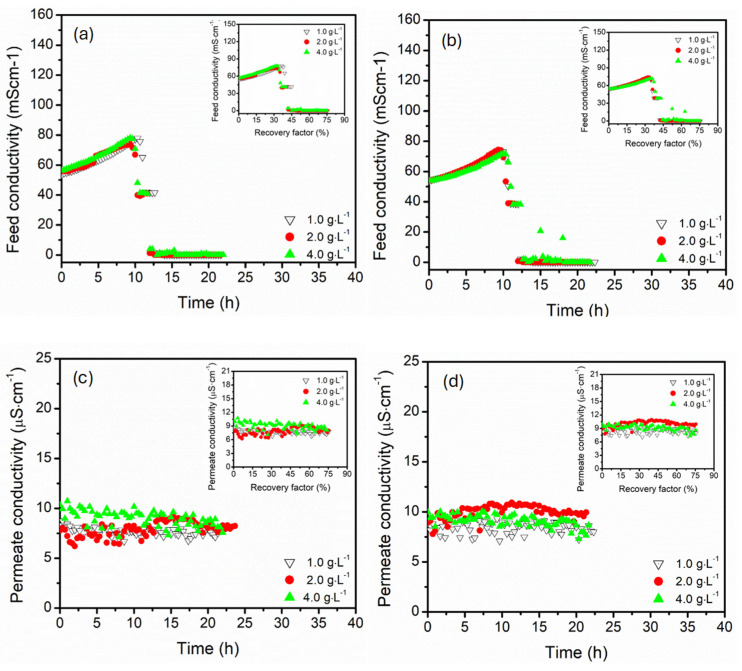
The effect of MgSO_4_ concentration on the feed conductivity profiles of the (**a**) parallel and (**b**) serial configurations and the permeate conductivity profiles of the (**c**) parallel and (**d**) serial configurations, all versus time in hollow fibre DCMD. Plot insert reflected the corresponding conductivity trends as a function of recovery factor. The feed and permeate circulation flow rate and feed temperature were kept constant at 27.5 mL·s^−1^ (0.547 m·s^−1^) and 60 °C, respectively. The DCMD was operated in a counter-current configuration.

The influence of MgSO_4_ concentration (1.0, 2.0, and 4.0 g·L^−1^) and module configuration (parallel and serial) on the feed and permeate conductivity response is presented in Figure 9. Here, the feed temperature (60 °C), flow rate (27.5 mL·s^−1^ and NaCl concentration (3.5 wt.%) were kept constant. A similar trend of rise in feed conductivity was recorded upon an increase in recovery factor, which was linked to water recovery from the feed. The feed conductivity decline became abrupt at high MgSO_4_ concentration, particularly in parallel configuration, suggesting immediate precipitation, which was linked to the feed solution exceeding the saturation index of MgSO_4_. In parallel connection, the pronounced decline was influenced by the localised supersaturation. The system experienced a relatively uniform concentration of the feed, leading to abrupt boundary layer crystallisation of MgSO_4_, which caused a sudden reduction in conductivity, particularly at high MgSO_4_ concentration (4.0 g·L^−1^). Conversely, the gradual decline in feed conductivity was reported in serial, indicating a delayed onset of precipitation. This was linked to a stepwise increase in feed concentration (noted from the slopes of the feed conductivity increase over time), promoting the progressive supersaturation. The permeate conductivity remained low (approximately ≤10.5 µS·cm^−1^) for both configurations under all tested MgSO_4_, demonstrating high salt rejection (>99.9%). The permeate conductivity remained stable even after salt precipitation events, confirming crystallisation occurrence in the bulk or boundary layer without affecting the membrane pores. The permeate conductivity was more stable in serial connection across all MgSO_4_ concentrations, particularly at 4.0 g·L^−1^. This suggested a reduced risk of supersaturation, with minimised wetting or ionic intrusion even at high recovery factors (>75%). On one hand, parallel connection experienced more variable permeate conductivity, particularly at high MgSO_4_ concentrations (2.0 and 4.0 g·L^−1^), suggesting the localised precipitation causing the temporal concentrations near the membrane interface [60]. The plot insert showed superimposed effects of MgSO_4_ concentrations (particularly 1.0 and 2.0 g·L^−1^), indicating the conductivity changes dominance by the dilution and concentration effects rather than precipitation. At high MgSO_4_ concentration (4.0 g·L^−1^), the feed concentration approaching the solubility limit presented the divergent trends, marking the influence of phase separation and scaling.

**Figure 10:**
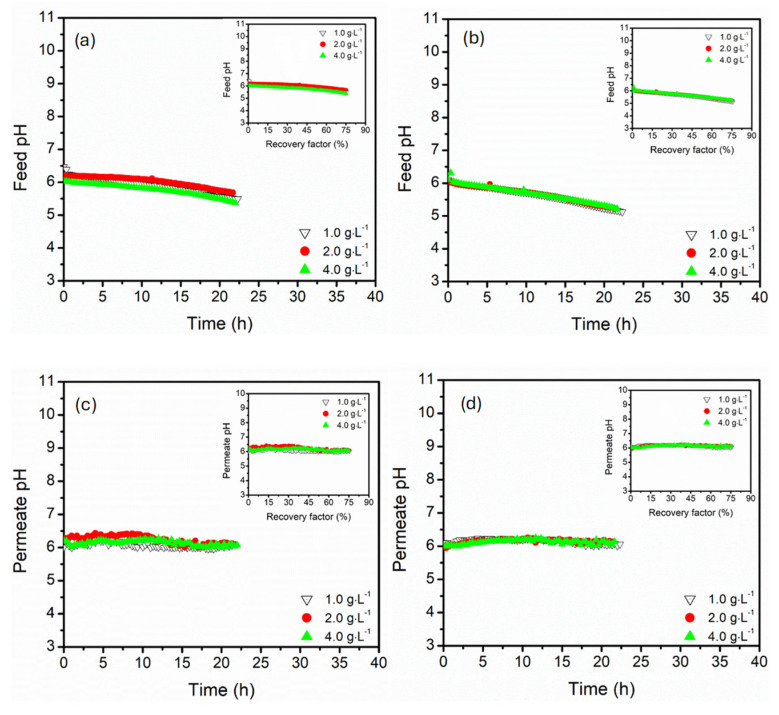
The pH profiles of the feed (**a**) parallel and (**b**) serial hollow fibre DCMD, permeate in (**c**) parallel and (**d**) serial hollow fibre DCMD configurations under varying MgSO_4_ (1.0, 2.0, and 4.0 g·L^−1^). Insert presented corresponding flux and ∆*T* as a function of recovery factor. The feed and permeate circulation flow rate and feed temperature were kept constant at 27.5 mL·s^−1^ (0.547 m·s^−1^) and 60 °C, respectively. The DCMD was operated in a counter-current configuration.

The influence of MgSO_4_ on the evolution of feed and permeate pH was assessed to understand the process salt behaviour, acid-base equilibrium, and membrane selectivity (Figure 10). The 3.5 wt.% feed solution doped with MgSO_4_ at concentrations of 1.0, 2.0, and 4.0 g·L^−1^ was processed in DCMD configured in parallel and serial configurations under constant feed temperature and circulation flow rate of 60 °C and 27.5 mL·s^−1^, respectively. The feed pH decreased consistently over time in both parallel and serial configurations, with values decreasing from 6.4 to 5.2. The feed pH declined steeply in serial connection, particularly pronounced at higher MgSO_4_ concentrations (4.0 g·L^−1^). This abrupt acidification in serially connected modules was associated with localised crystallisation of MgSO_4_ phases such as epsomite, which consumed water buffering capacity and released H^+^, resulting in a pH decrease [61]. On one hand, the parallel configuration presented a gradual and progressive decline in pH, particularly at lower MgSO_4_ concentrations (1.0 and 2.0 g·L^−1^), indicating the evenly distributed effects of concentrations across all modules. The parallel configuration promoted uniform hydraulic conditions, limiting the localised supersaturation and slowing the pH shifts. The permeate pH remained relatively stable (~6.2–6.5) across all conditions, even at high recovery factors. This consistently confirmed the high selectivity of the hydrophobic PP membrane, successfully rejecting the charged ionic species. The permeate pH was more stable in the serial configuration for all MgSO_4_ concentrations, indicating the effective ionic species and water separation across the module train. However, minor pH fluctuations were recorded in parallel configurations, particularly at high MgSO_4_ concentration (4.0 g·L^−1^), reflecting the variations in micro-scale and transient precipitation dynamics at the membrane interface.

### 3.4. Effect of Flow Direction (Co-Current vs. Counter-Current)

**Figure 11:**
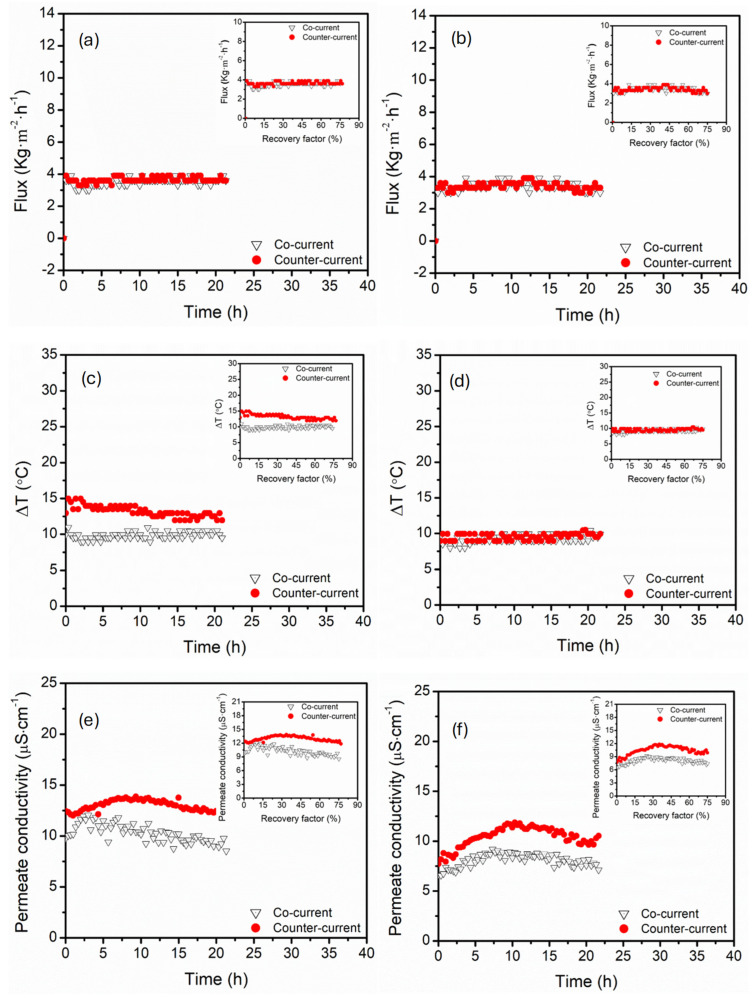
The influence of co-current and counter-current on process performance of MD modules configured in (**a**,**c**,**e**) parallel and (**b**,**d**,**f**) serial connections. Panels (**a**,**b**) permeate flux, (**c**,**d**) ∆*T*, (**e**,**f**) permeate conductivity. The following process conditions were kept constant: feed temperature (60 °C), flow rate (27.5 mL·s^−1^), feed composition (3.5 wt% NaCl, 2.0 g·L^−1^ MgSO_4_). Plot insert is the same data plotted against the recovery factor instead of time.

The relative flow direction of the feed and permeate plays an important role in MD systems. The co-current (same direction) and counter-current (opposite directions) influence the heat and mass transfer dynamics differently [21,62]. Although theoretical models indicated temperature polarisation reductions in counter-current flow, limited empirical data on this flow in different configurations (parallel and serial) motivated for evaluation in the current study [63]. Across all module configurations and flow orientations, flux remained relatively similar (Figure 11). The minor flux fluctuations were relatively superimposed between co- and counter-current flow directions in serial configurations, possibly due to a staged thermal gradient, which buffered the effects of the modules in series. In parallel configuration, the co-current presented greater fluctuations due to the rapid collapse of the thermal gradient induced by different feed inlets [22]. Though the permeate flux was superimposed for all module configurations and flow orientations, counter-current flow produced high ∆*T* in the parallel configuration, suggesting a high driving force enabled by the opposing feed and permeate flow, which reduced thermal equilibrium between adjacent fluids. ∆*T* remained superimposed in serial configurations under both co- and counter-current flow. This was influenced by cascaded thermal profiles, which enforced the normally distributed ∆*T* along serial modules, resulting in less sensitivity to flow directions. The permeate conductivity of both module configurations remained relatively small under co- and counter-current conditions, which declined further at high recovery factors, suggesting high process selectivity and low risk of membrane wetting. The permeate conductivity was maintained below 13.6 µS·cm^−1^ under all tested conditions, confirming process salt rejection > 99.9%. Although the permeate conductivity was relatively low at high recovery factors, co-current flow exhibited a clear increase in permeate conductivity over time (more pronounced at low recovery factors ≤ 30%). This was associated with localised temperature polarisation and back-diffusion of ions.

### 3.5. Thermal Efficiency of Hollow Fibre DCMD Under Varying Operational Conditions in Different Module Connection Configurations

The thermal efficiency, complementing and reinforcing the findings presented in the previous sections, is presented in Table 2. The following relationship was used to calculate thermal efficiency (*TE*).

$$TE=\frac{Q_{evap}}{Q_{feed}}=\frac{J\cdot A\cdot \Delta H_{evap}}{m\cdot C_{p}\cdot \Delta T}$$

where

*J* is the distillation flux (kg·m^−2^·s^−1^)

*A* is the active membrane area (m^2^)

∆*H_evap_* is the latent heat of vaporisation (kJ·kg^−1^)

*m* is the feed mass flow rate (kg·s^−1^)

*C_p_* is the specific heat capacity (kJ·kg^−1^·K^−1^)

∆*T* is the feed temperature across the membrane (K)

The thermal efficiency (*TE*) declined from 26.4% at 50 °C to 21.7% at 70 °C in a serial connection configuration, suggesting intensified temperature polarisation at high temperatures. This was consistent with the reported decrease in ∆*T* along the module. The decreased ∆*T* reduced the thermal energy input required for evaporation. Parallel configuration retained high thermal efficiency (30.8% to 22.3%), reflecting even distribution of the heat across the modules, with less thermal degradation compared with the serial configuration. This was ascertained by stable ∆*T* profiles reported previously. In the case of varied circulation flow rates, the thermal efficiency remained relatively stable across all tested flow rates, ranging from 25.1% to 26.1% in the serial configuration. However, the parallel configuration presented fluctuating thermal degradation with thermal efficiency of 21.0% at 27.5 mL·s^−1^. These trends aligned with stabilised ∆*T*, though declines were recorded beyond certain thresholds, demonstrating hydrodynamic inefficiencies. Upon increasing the concentration of MgSO_4_, the permeate flux decreased slightly, particularly in the serial configuration. Similarly, the thermal efficiency decreased at high MgSO_4_ loading. Specifically, the thermal efficiency decreased from 28.9% to 20.8% in the serial configuration and from 32.9% to 30.0% in the parallel configuration. Based on these trends, the heat transfer was reduced due to increased resistance at the membrane interface, which was caused by concentration polarisation or early-stage scale formation. Though no direct wetting was assessed, the sharp decline in thermal efficiency at 4.0 g·L^−1^ suggested salt accumulation, which impaired evaporation dynamics. Based on Figure 11, the co-current feed and permeate circulation presented stable fluxes, particularly in the parallel configuration. This was supported by the thermal efficiency data, where the co-current configuration achieved the highest efficiency of 32.9% compared to 30.0% (counter-current) in the parallel configuration. Similarly, the co-current circulation achieved thermal efficiency of 34.0% compared to counter-current (28.9%) in the serial configuration. These results suggested that under the current design, co-current circulation provided resistance to thermal losses, thus indicating its direct influence on thermal gradient stability.

**Table 2:** The influence of varying process parameters and module configurations on the thermal efficiency of DCMD.

Process Conditions	Connection Configuration	Parameters	Thermal Efficiency (%)
Effect of feed temperature (°C)	Serial	50	26.4
		60	24.2
		70	21.7
	Parallel	50	30.8
		60	20.5
		70	22.3
Effect of circulation flow rate (mL·s^−1^)	Serial	22.1	25.1
		27.5	26.4
		32.3	26.1
	Parallel	22.1	27.9
		27.5	21.0
		32.3	22.0
Effect of MgSO_4_ concentration (g·L^−1^)	Serial	1.0	28.9
		2.0	27.8
		4.0	20.8
	Parallel	1.0	32.0
		2.0	25.5
		4.0	19.6
Circulation flow configuration	Serial	Co-current	34.0
		Counter-current	28.9
	Parallel	Co-current	32.9
		Counter-current	32.0

## 4. Conclusions

The current study presented the holistic multiparameter evaluation of parallel and serial module connection configurations for water desalination in hollow fibre direct contact membrane distillation (DCMD). Assessed key operating variables were feed temperature, feed/permeate flow rates, concentrations of magnesium sulphate (MgSO_4_, scalant) and fluid flow directions to understand their hydrodynamic and thermal influence on the process performance and stability. Although both configurations achieved high salt rejection (99.9%) across all tested process conditions, their operational dynamics were influenced differently.

- Parallel configuration exhibited high operational stability, controlled polarisation effects, and delayed flux decline, even at high recovery factors (74%). This process architecture enhanced hydrodynamic distributions with supported uniform thermal and mass transfer, suggesting maintenance of long-term stable flux even at high recovery factors.
- In contrast, the serial configuration presented intense supersaturation at lower recovery factors, facilitating the earlier onset of crystallisation. Despite the great advantage of serial configuration in crystallisation processes, the process suffered from thermal and concentration polarisation, particularly at elevated temperatures and low flow rates, causing flux instabilities and the risk of scaling along the sequential module path, especially in long-term operations. Upon increasing the circulation flow rates, thermal efficiency was maintained in this configuration, indicating resistance to thermal losses.

Based on assessed conductivity and pH profiles, both configurations demonstrated resistance to performance deterioration under all tested conditions. Salt crystallisation occurred predominantly in the bulk of the feed water or boundary layer without direct influence on performance loss, particularly in the parallel configuration. The investigated multiparameter influence on parallel and serial module configurations provided an insight into how the process architecture governs the mass and heat transfers in DCMD systems. These findings provided the guide for MD module configuration and parameter optimisation design for high recovery in water desalination, brine concentration, and sustainable water reuse, reinforcing the potential of DCMD in thermal-driven desalination strategies and circular water economics.

## Figures and Tables

**Table 1:** Feed and permeate inlet and outlet temperatures corresponding to 50, 60, and 70 °C set temperatures. The inlet temperature in the parallel configuration refers to the feed temperature supplied to both modules simultaneously, while in the serial configuration, the inlet temperature is the one entering the first module. Similarly, the outlet temperature refers to the temperature exiting both modules in parallel configuration and the last module in serial configuration.

Connection Configuration	Feed Temperature (°C)	Feed Inlet Temperature (°C)	Feed Outlet Temperature (°C)	Permeate Inlet Temperature (°C)	Permeate Outlet Temperature (°C)
Serial	50	33.2	29.1	19.9	23.1
	60	37.8	34.2	23.1	26.2
	70	44.3	38.8	23.6	28.5
Parallel	50	32.5	28.7	20.1	23.3
	60	37.2	33.8	23.2	27.1
	70	43.5	37.9	24.1	29.7

## Data Availability

The authors declare that the data supporting the current findings of this study are available within the manuscript. Should any raw data be required, please contact the corresponding author.

## References

[1] He C., Liu Z., Wu J., Pan X., Fang Z., Li J., Bryan B.A. (2021). Future global urban water scarcity and potential solutions. Nat. Commun..

[2] Salehi M. (2022). Global water shortage and potable water safety; Today’s concern and tomorrow’s crisis. Environ. Int..

[3] Mcdonald R.I., Green P., Balk D., Fekete B.M., Revenga C., Todd M., Montgomery M. (2011). Urban growth, climate change, and freshwater availability. Proc. Natl. Acad. Sci. USA.

[4] Wee S.Y., Aris A.Z. (2023). Revisiting the “forever chemicals”, PFOA and PFOS exposure in drinking water. npj Clean Water.

[5] Lee B.X., Kjaerulf F., Turner S., Cohen L., Donnelly P.D., Muggah R., Davis R., Realini A., Kieselbach B., MacGregor L.S. (2016). Transforming Our World: Implementing the 2030 Agenda Through Sustainable Development Goal Indicators. J. Public Health Policy.

[6] van Vliet M.T.H., Jones E.R., Flörke M., Franssen W.H.P., Hanasaki N., Wada Y., Yearsley J.R. (2021). Global water scarcity including surface water quality and expansions of clean water technologies. Environ. Res. Lett..

[7] Alenezi A., Alabaiadly Y. (2025). Emerging technologies in water desalination: A review and future outlook. Energy Nexus.

[8] Moreira V.R., Raad J.V., Lazarini J.X., Santos L.V.S., Amaral M.C.S. (2023). Recent progress in membrane distillation configurations powered by renewable energy sources and waste heat. J. Water Process Eng..

[9] Xu H., Zhang Q., Song N., Chen J., Ding M., Mei C., Zong Y., Chen X., Gao L. (2022). Membrane distillation by novel Janus-enhanced membrane featuring hydrophobic-hydrophilic dual-surface for freshwater recovery. Sep. Purif. Technol..

[10] Gryta M. (2012). Effectiveness of water desalination by membrane distillation process. Membranes.

[11] Nthunya L.N., Bopape M.F., Mahlangu O.T., Mamba B.B., Van der Bruggen B., Quist-Jensen C.A., Richards H. (2022). Fouling, performance and cost analysis of membrane-based water desalination technologies: A critical review. J. Environ. Manag..

[12] Gontarek-castro E., Castro-Muñozb R. (2024). How to make membrane distillation greener: A review of environmentally friendly and sustainable aspects. Green Chem..

[13] Abdelgaied M., Seleem M.F., Bassuoni M.M. (2022). Recent technological advancements in membrane distillation and solar stills: Preheating techniques, heat storage materials, and nanomaterials—A detailed review. Environ. Sci. Pollut. Res..

[14] Zaragoza G.A., Andrés-Mañas J., Ruiz-Aguirre A. (2018). Commercial scale membrane distillation for solar desalination. npj Clean Water.

[15] Yadav A., Labhasetwar P.K., Shahi V.K. (2021). Membrane distillation using low-grade energy for desalination: A review. J. Environ. Chem. Eng..

[16] Ho C., Chen L., Lim J., Lin P., Lu P. (2021). Distillate Flux Enhancement of Direct Contact Membrane Distillation Modules with Inserting Cross-Diagonal Carbon-fiber Spacers. Membranes.

[17] Ezugbe E.O., Rathilal S. (2020). Membrane technologies in wastewater treatment: A review. Membranes.

[18] Nthunya L.N., Chong K.C., Lai S.O., Lau W.J., López-Maldonado E.A., Camacho L.M., Shirazi M.M.A., Ali A., Mamba B.B., Osial M. (2024). Progress in membrane distillation processes for dye wastewater treatment: A review. Chemosphere.

[19] Tai Z.S., Othman M.H.D., Koo K.N., Jaafar J. (2023). Critical review on membrane designs for enhanced flux performance in membrane distillation. Desalination.

[20] Liu J., Xie B., Mushtaq N., Xu G., Bar-Zeev E., Hu Y. (2024). New insights into the role of carbon nanotubes spray-coated on both sides of the PTFE membrane in suppressing temperature polarization and enhancing water flux in direct contact membrane distillation. J. Membr. Sci..

[21] Ali A., Shirazi M.M.A., Nthunya L., Castro-Muñoz R., Ismail N., Tavajohi N., Zaragoza G., Quist-Jensen C.A. (2024). Progress in module design for membrane distillation. Desalination.

[22] Camacho L.M., Dumée L., Zhang J., de Li J., Duke M., Gomez J., Gray S. (2013). Advances in membrane distillation for water desalination and purification applications. Water.

[23] Chen L., Xu P., Wang H. (2020). Interplay of the factors affecting water flux and salt rejection in membrane distillation: A state-of-the-art critical review. Water.

[24] Ho C.D., Wang Y.W., Chao Y., Chew T.L., Jiang M.S., Chen J.H., Li C.Y. (2024). Enhancing the Permeate Flux Improvement of Direct Contact Membrane Distillation Modules with Inserted S-Ribs Carbon-Fiber Filaments. Membranes.

[25] Francis L., Ahmed F.E., Hilal N. (2022). Advances in Membrane Distillation Module Configurations. Membranes.

[26] Lawson K.W., Lloyd D.R. (2021). Membrane distillation. J. Membr. Sci..

[27] Lawson K.W., Lloyd D.R. (1996). Membrane distillation. I. Module design and performance evaluation using vacuum membrane distillation. J. Membr. Sci..

[28] Andersson S.-I., Kjellander N., Rodesjö B. (1985). Design and field tests of a new membrane distillation desalination process. Desalination.

[29] Bin Abid M., Wahab R.A., Salam M.A., Moujdin I.A., Gzara L. (2023). Desalination technologies, membrane distillation, and electrospinning, an overview. Heliyon.

[30] Khalifa A.E., Alawad S.M. (2018). Air gap and water gap multistage membrane distillation for water desalination. Desalination.

[31] Alawad S.M., Khalifa A.E. (2021). Case Studies in Thermal Engineering Development of an efficient compact multistage membrane distillation module for water desalination. Case Stud. Therm. Eng..

[32] Alawad S.M., Khalifa A.E. (2021). Performance and energy evaluation of compact multistage air gap membrane distillation system: An experimental investigation. Sep. Purif. Technol..

[33] Aryapratama R., Koo H., Jeong S., Lee S. (2016). Performance evaluation of hollow fiber air gap membrane distillation module with multiple cooling channels. Desalination.

[34] Ali A., Aimar P., Drioli E. (2015). Effect of module design and flow patterns on performance of membrane distillation process. Chem. Eng. J..

[35] Meindersma G.W., Guijt C.M., de Haan A.B. (2006). Desalination and water recycling by air gap membrane distillation. Desalination.

[36] Kariman H., Mohammed H.A., Zargar M., Khiadani M. (2025). Performance comparison of flat sheet and hollow fibre air gap membrane distillation: A mathematical and simulation modelling approach. J. Membr. Sci..

[37] Dong Y., Dai X., Zhao L., Gao L., Xie Z., Zhang J. (2021). Review of transport phenomena and popular modelling approaches in membrane distillation. Membranes.

[38] Herwig H. (2016). What exactly is the Nusselt number in convective heat transfer problems and are there alternatives?. Entropy.

[39] Cancilla N., Tamburini A., Tarantino A., Visconti S., Ciofalo M. (2022). Friction and Heat Transfer in Membrane Distillation Channels: An Experimental Study on Conventional and Novel Spacers. Membranes.

[40] Ali E. (2022). Optimal Control of Direct Contact Membrane Distillation Operated under Fluctuating Energy Source. Membranes.

[41] Nthunya L.N., Ali A., Richards H., Chimuka L., Quist-Jensen C., Mamba B.B. (2025). Innovative dual-purpose remediation of acid mine drainage and resource recovery through membrane distillation crystallization. npj Clean Water.

[42] Nthunya L.N., Setati B., Richards H., Chimuka L., Mamba B.B. (2025). From extract to crystals: Unlocking green nanotechnology towards recovery of bioactive compounds from Moringa oleifera via membrane distillation crystallization. Sep. Purif. Technol..

[43] McGaughey A.L., Childress A.E. (2022). Wetting indicators; modes, and trade-offs in membrane distillation. J. Membr. Sci..

[44] Silva R., Cadorin L., Rubio J. (2010). Sulphate ions removal from an aqueous solution: I. Co-precipitation with hydrolysed aluminum-bearing salts. Miner. Eng..

[45] Tomaszewska M., Gryta M., Morawski A. (1995). Study on the concentration of acids by membrane distillation. J. Membr. Sci..

[46] Wang F., Liu J., Li D., Liu Z., Zhang J., Ding P., Liu G., Feng Y. (2022). High-Efficiency Water Recovery from Urine by Vacuum Membrane Distillation for Space Applications: Water Quality Improvement and Operation Stability. Membranes.

[47] Alkhatib A., Ayari M.A., Hawari A.H. (2021). Fouling mitigation strategies for different foulants in membrane distillation. Chem. Eng. Process. Process. Intensif..

[48] Moujdin I.A., Totah H.S., Allaf I., Abulkhair H., Fayed M. (2025). Evaluation of swirling flow phenomenon in direct contact membrane distillation. Front. Mech. Eng..

[49] Ve Q.L., Rahaoui K., Bawahab M., Faqeha H., Date A., Akbarzadeh A., Do M.C., Nguyen Q.L., Ve L., Do C. (2019). Experimental Investigation of Heat Transfer Correlation for Direct Contact Membrane Distillation. J. Heat Transf..

[50] Ali K., Abdelsamie M.M., Ali M.I.H. (2024). Toward sustainable energy resource: Integrated concentrated photovoltaic and membrane distillation systems for fresh water and electricity production. Energy.

[51] Blankert B., Vrouwenvelder J.S., Witkamp G.-J., Ghaffour N. (2020). Minimum net driving temperature concept for membrane distillation. Membranes.

[52] Abu-Zeid M.A.E.-R., Lu X., Zhang S. (2020). Developing symmetrical traditional double-stage membrane distillation system based on air-gap and water-gap configurations. Desalination Water Treat..

[53] Chimanlal I., Nthunya L.N., Quist-Jensen C., Richards H. (2022). Membrane distillation crystallization for water and mineral recovery: The occurrence of fouling and its control during wastewater treatment. Front. Chem. Eng..

[54] Nthunya L.N., Pinier J., Ali A., Quist-jensen C., Richards H. (2024). Valorization of acid mine drainage into potable water and valuable minerals through membrane distillation crystallization. Sep. Purif. Technol..

[55] Balis E., Griffin J.C., Hiibel S.R. (2022). Membrane Distillation-Crystallization for inland desalination brine treatment. Sep. Purif. Technol..

[56] Li X., Pan J., Macedonio F., Ursino C., Carraro M., Bonchio M., Drioli E., Figoli A., Wang Z., Cui Z. (2022). Fluoropolymer Membranes for Membrane Distillation and Membrane Crystallization. Polymers.

[57] Rezaei M., Warsinger D.M., Duke M.C., Matsuura T., Samhaber W.M. (2018). Wetting phenomena in membrane distillation: Mechanisms, reversal, and prevention. Water Res..

[58] Nthunya L.N., Gutierrez L., Lapeire L., Verbeken K., Zaouri N., Nxumalo E.N., Mamba B.B., Verliefde A.R., Mhlanga S.D. (2019). Fouling resistant PVDF nanofibre membranes for the desalination of brackish water in membrane distillation. Sep. Purif. Technol..

[59] Schilling S., Glade H. (2023). Review and Analysis of Heat Transfer in Spacer-Filled Channels of Membrane Distillation Systems. Membranes.

[60] Khalifa A.E., Alawad S.M., Antar M.A. (2017). Parallel and series multistage air gap membrane distillation. Desalination.

[61] Chou I.-M., Seal R.R. (2003). Determination of Epsomite-Hexahydrite Equilibria by the Humidity-Buffer Technique at 0.1 MPa with Implications for Phase Equilibria in the System MgSO_4_-H_2_O. Astrobiology.

[62] Soumbati Y., Bouatou I., Abushaban A., Belmabkhout Y., Necibi M.C. (2025). Review of membrane distillation for desalination applications: Advanced modeling, specific energy consumption, and water production cost. J. Water Process. Eng..

[63] Meo R.R., Craveri L., Bertozzi E., Malaguti M., Tiraferri A., Morciano M., Fasano M. (2025). Systematic exploration of direct solar absorption potential to enhance direct contact membrane distillation. Desalination.

